# *N*,*N*-disubstituted azines attenuate LPS-mediated neuroinflammation in microglia and neuronal apoptosis via inhibiting MAPK signaling pathways

**DOI:** 10.1186/s12868-017-0399-3

**Published:** 2017-12-28

**Authors:** Lalita Subedi, Oh Wook Kwon, Chaeho Pak, Goeun Lee, Kangwoo Lee, Hakwon Kim, Sun Yeou Kim

**Affiliations:** 1 College of Pharmacy, Gachon University, #191, Hambakmoero, Yeonsu-gu, Incheon, 21936 Republic of Korea; 2 Graduate School of East-West Medical Science, Kyung Hee University, Global Campus, #1732 Deogyeong-daero, Giheung-gu, Yongin, Gyeonggi-do 446-701 Republic of Korea; 3 Department of Applied Chemistry and Institute of Natural Sciences, Kyung Hee University, Global Campus, #1732 Deogyeong-daero, Giheung-gu, Yongin, Gyenggi-do 446-701 Republic of Korea

**Keywords:** Azine, Lipopolysaccharide, Neuroinflammation, MAPK, Apoptosis

## Abstract

**Background:**

Activated microglia interact with astrocytes and neuronal cells to induce neuroinflammation, which can contribute to the pathogenesis and progression of Alzheimer’s and Parkinson’s disease. To identify the most effective anti-neuroinflammatory agent, we designed and synthesized a family of 13 new azine derivatives and investigated their anti-neuroinflammatory activities in LPS-activated BV-2 microglial cells.

**Results:**

Out of 13 derivatives, **compound 3** [4,4′-(1*E*,1′*E*,3*E*,3′*E*)-3,3′-(hydrazine-1,2-diylidene) bis-(prop-1-ene-1-yl-3-ylidene) bis-(2-methoxyphenol)] exhibited excellent anti-neuroinflammatory activities (IC_50_ = 12.47 µM), which protected neurons from microglia-mediated neurotoxicity. Specifically, the anti-neuroinflammatory effects of **compound 3** inhibited MAPK signaling pathways through the inhibition of p38 and JNK mediated signaling and the production of pro-inflammatory cytokines, and inflammatory mediators. Additionally, **compound 3** strongly exhibited neuroprotective effect by inhibiting LPS-mediated necrosis and apoptosis. Preliminary SAR analysis suggests that the presence of methoxyphenol and the substitution pattern within hydrazine may influence the anti-neuroinflammatory activity. FACS analysis also strongly supports the neuroprotective effect of **compound 3**.

**Conclusions:**

Based on our results, the **compound 3** exhibited excellent anti-neuroinflammatory activity against LPS-activated microglia, which resulted in the inhibition of neuronal apoptosis and neuronal degeneration.

**Electronic supplementary material:**

The online version of this article (10.1186/s12868-017-0399-3) contains supplementary material, which is available to authorized users.

## Background

Microglia are the resident immune cells that play a crucial role in the innate immune response in the normal brain and central nervous system (CNS). However, chronic neuroinflammation induced by activated microglia is the major cause of neurodegenerative diseases like Alzheimer’s disease (AD) and Parkinson’s disease (PD) [1–3]. Determining the regulators of neuroinflammation is very important for the treatment of neurodegenerative diseases. Extracellular stimuli such as lipopolysaccharide (LPS), and interferon-γ (IFN-γ) can activate microglia and secrete pro-inflammatory mediators, which will initiate several major cellular responses that contribute to the pathogenesis of neuroinflammation [4–7]. Pro-inflammatory mediators such as nitric oxide (NO), prostaglandin E2 (PGE2), interleukin-6 (IL-6), interleukin-1β (IL-1β), and tumor necrosis factor-α (TNF-α) are released by activated microglia and will further activate astrocytes, and vice versa. Although the normal activation of microglia and astrocytes is critical for the health of the CNS, chronic activation of microglia and astrocytes can induce neuronal toxicity and cell death. Upon activation, microglial cells grow large, assume an amoeboid shape, and initiate the inflammatory cascades in the brain [8–10].

Chronic injury and toxins with chronic inflammation can eventually lead to neurodegeneration. LPS, as TLR4 agonist, can activate the receptor followed by an increase in the phosphorylation of MAPK signaling proteins, and can further activate nuclear factor κB (NF-κB) mediated transcription of various inflammatory factors like inducible nitric oxide synthase (iNOS), NO, cyclooxygenase 2 (COX-2), PGE2, arachidonic acid, eicosanoids, and reactive oxygen species (ROS). In addition, chronic inflammation can increase the levels of proinflammatory cytokines such as TNF-α, IL-6, and IL-1β. NO, biosynthesized from l-arginine by inducible nitric oxide synthase (iNOS) plays an important role in the modulation of inflammatory responses [11]. Similarly, iNOS is also an important enzyme regulator of neuroinflammation [12–14]. PGE2 production is catalyzed by COX-2, which is a key neuroinflammatory enzyme that controls the immune system and ascending pain pathway in the brain [15]. Furthermore, LPS-induced inflammatory cytokines play a major role in the brain immune system. LPS-activated microglia, inflammatory mediators, and pro-inflammatory cytokines, such as activated astrocytes, can further increase inflammatory cascades by inducing the activation of ramified microglia. This negative crosslinking of microglia and astrocytes induce the degeneration and death of neurons. AD and PD are characterized by such conditions [16].

Murine BV-2 microglia cells exhibit morphological, functional, and phenotypical properties similar to primary microglia. Therefore, BV-2 microglial cells are the perfect alternative to primary microglia in which to study the various microglial responses and interactions in vitro. Several of our previous studies demonstrated the anti-inflammatory or protective effects of natural derivatives in LPS-stimulated BV-2 cells. For example, 6-shogaol modulated neuroinflammation [17], and wogonin, a flavone from the root of *Scutellaria baicalensis*, protected the brain from oxygen and glucose deprivation [18].

Azine have been reported to have biological actions such as antiviral effects, anticonvulsant activity [19, 20], and histamine H4 receptor antagonistic activity [21, 22]. Several pharmacological studies have suggested the potential utility of histamine H4R antagonists/inverse agonists in the treatment of inflammatory diseases such as allergic rhinitis, asthma, atopic dermatitis, colitis, and pruritus. However, no anti-neuroinflammatory studies on azine derivatives have been performed that specifically address the regulation of activated microglia. In this work, we report the synthesis of *N*,*N*-di-substituted azine derivatives and their anti-neuroinflammatory and neuroprotective effects in vitro.

## Methods

### Chemicals and reagents

Chemicals used in cell culture experiments including Dulbecco’s modified Eagle medium (DMEM), fetal bovine serum (FBS), and penicillin–streptomycin were obtained from Invitrogen (Carlsbad, CA, USA). LPS, N-monomethyl-l-arginine (NMMA). Enzyme link immune sorbent assay (ELISA) development kit, TNF-α (DY410), IL-6 (DY406), and PGE-2 (514010). Primary and secondary antibodies for iNOS (610333), COX-2 (Sc1745), pERK (9101s), ERK (9102s), pJNK (9251s), JNK (4671s), pp38 (9211s), p38 (9212s), and tubulin (T5168) were purchased from Santa Cruz (Capitola Rd, Santa Cruz, CA, USA), and Cell Signaling (Beverly, MA, USA) respectively. All other chemicals and reagents were purchased from Sigma Chemical (St. Louis, MO) unless otherwise stated.

### General procedure for azines

**Scheme 1:**
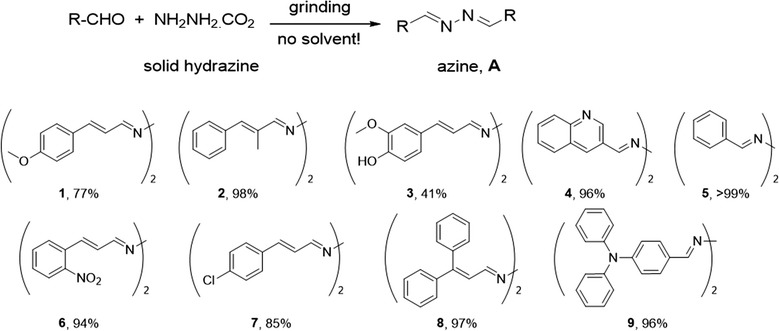
Synthetic schemes for azine derivatives

A vial containing aldehyde (1 mM) and solid hydrazinium carboxylate (0.5 mM) was heated at 60–80 °C in an oven until the reaction was complete. Subsequently, the generated water was removed by evaporation. A high yield of the corresponding azine was produced. The azine was used in the next reaction without further purification (Scheme 1).

#### 1,2-Bis((*E*)-3-(4-methoxyphenyl)allylidene)hydrazine (**1**)

Yield: 77.5%; ^1^H NMR: (300 MHz, CDCl_3_) *δ* 8.35 (*d*, *J* = 8.8 Hz, 2H), 7.47 (*d*, *J* = 8.8 Hz, 4H), 6.90–7.01 (*m*, 8H), 3.84 (*s*, 6H); ^13^C NMR: (75 MHz, CDCl_3_) *δ* 163.6, 160.7, 142.8, 128.9, 128.7, 123.2, 114.3, 55.3; LC–MS (ESI): 321.1 (M + 1).

#### 1,2-Bis((*E*)-2-methyl-3-phenylallylidene)hydrazine (**2**)

Yield: 98%; ^1^H NMR: (300 MHz, CDCl_3_) *δ* 8.35 (*s*, 2H), 7.39–7.53 (*m*, 10H), 6.91 (*s*, 2H), 2.27 (*s*, 6H); ^13^C NMR: (75 MHz, CDCl_3_) *δ* 166.3, 140.3, 136.5, 134.9, 129.5, 128.3, 127.9, 13.3; LC–MS (ESI): 289.1 (M + 1).

#### 4,4′-(1*E*,1′*E*,3*E*,3′*E*)-3,3′-(hydrazine-1,2-diylidene)bis(prop-1-ene-1-yl-3-ylidene)bis(2-methoxyphenol) (**3**)

Yield: 41.1%; ^1^H NMR: (300 MHz, CDCl_3_) *δ* 8.35 (*d*, *J* = 8.8 Hz, 2H), 6.89–7.06 (*m*, 10H), 5.82 (br s, 2H), 3.93 (*s*, 6H); ^13^C NMR: (75 MHz, CDCl_3_) *δ* 163.5, 147.3, 146.8, 143.2, 128.5, 123.1, 122.3, 114.7, 108.5, 55.8; LC–MS (ESI): 353.2 (M + 1).

#### 1,2-Bis(quinolin-3-ylmethylene)hydrazine (**4**)

Yield: 96%; ^1^H NMR: (300 MHz, CDCl_3_) *δ* 9.51 (*s*, 2H), 8.93 (*s*, 2H), 8.53 (*s*, 2H), 8.18 (*d*, *J* = 8.4 Hz, 2H), 7.95 (*d*, *J* = 8.1 Hz, 2H), 7.82 (*t*, *J* = 7.7 Hz, 2H), 7.64 (*t*, *J* = 7.3 Hz, 2H); ^13^C NMR: (75 MHz, CDCl_3_) *δ* 160.4, 149.5, 149.2, 136.9, 131.0, 129.6, 128.6, 127.5, 127.4, 126.9; LC–MS (ESI): 311.1 (M + 1).

#### 1,2-Dibenzylidenehydrazine (**5**)

Yield: 99.8%; ^1^H NMR: (300 MHz, CDCl_3_) *δ* 8.68 (*s*, 2H), 7.83–7.86 (*m*, 4H), 7.45–7.47 (*m*, 6H); ^13^C NMR: (75 MHz, CDCl_3_) *δ* 162.0, 134.0, 131.1, 128.7, 128.5; LC–MS (ESI): 209.1 (M + 1).

#### 1,2-Bis((*E*)-3-(2-nitrophenyl)allylidene)hydrazine (**6**)

Yield: 93.8%; ^1^H NMR: (300 MHz, CDCl_3_) *δ* 8.40 (*d*, *J* = 8.8 Hz, 2H), 8.04 (*d*, *J* = 8.1 Hz, 2H), 7.77 (*d*, *J* = 7.7 Hz, 2H), 7.70 (*d*, *J* = 7.32 Hz, 2H), 7.63 (*d*, *J* = 15.4 Hz, 2H), 7.52 (*t*, *J* = 7.7 Hz, 2H), 7.02–7.11 (*m*, 2H); ^13^C NMR: (75 MHz, CDCl_3_) *δ* 163.1, 148.0, 137.8, 133.4, 131.2, 130.1, 129.7, 128.4, 125.0; LC–MS (ESI): 350.1 (M + 1).

#### 1,2-Bis((*E*)-3-(4-chlorophenyl)allylidene)hydrazine (**7**)

Yield: 85%; ^1^H NMR: (300 MHz, CDCl_3_) *δ* 8.36 (*t*, *J* = 4.4 Hz, 2H), 7.46 (*d*, *J* = 8.8 Hz, 4H), 7.36 (*d*, *J* = 8.8 Hz, 4H), 7.05 (*d*, *J* = 4.1 Hz, 4H); ^13^C NMR: (75 MHz, CDCl_3_) *δ* 163.5, 141.9, 135.3, 134.2, 129.2, 128.5, 125.9; LC–MS (ESI): 329.0 (M^+^), 331.0 (M + 2).

#### 1,2-Bis(3,3-diphenylallylidene)hydrazine (**8**)

Yield: 97%; ^1^H NMR: (300 MHz, CDCl_3_) *δ* 8.25 (*d*, *J* = 10.2 Hz, 2H), 7.23–7.43 (*m*, 20H), 6.97 (*d*, *J* = 10.3 Hz, 2H); ^13^C NMR: (75 MHz, CDCl_3_) *δ* 162.4, 154.1, 140.8, 138.2, 130.3, 129.1, 128.4, 128.1, 124.1; LC–MS (ESI): 413.2 (M + 1).

#### 4,4′-Hydrazine-1,2-diylidenebis(methan-1-yl-1-ylidene)bis(*N*,*N*-diphenylaniline) (**9**)

Yield: 96%; ^1^H NMR: (300 MHz, CDCl_3_) *δ* 8.59 (*s*, 2H), 7.66 (*d*, *J* = 8.8 Hz, 4H), 7.27–7.33 (*m*, 8H), 7.05–7.16 (*m*, 16H); ^13^C NMR: (75 MHz, CDCl_3_) *δ* 160.9, 150.4, 147.0, 129.5, 129.4, 127.3, 125.4, 123.9, 121.7; LC–MS (ESI): 543.2 (M + 1).

**Fig. 1:**
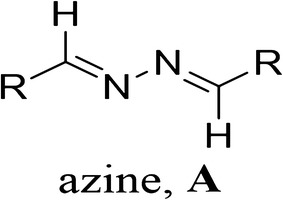
General structure of azine

We designed and synthesized several derivatives of azine using the solvent-free reaction of aldehyde with solid hydrazine to produce the corresponding azine [23–25] (Fig. 1).

### Cell culture

**Scheme 2:**
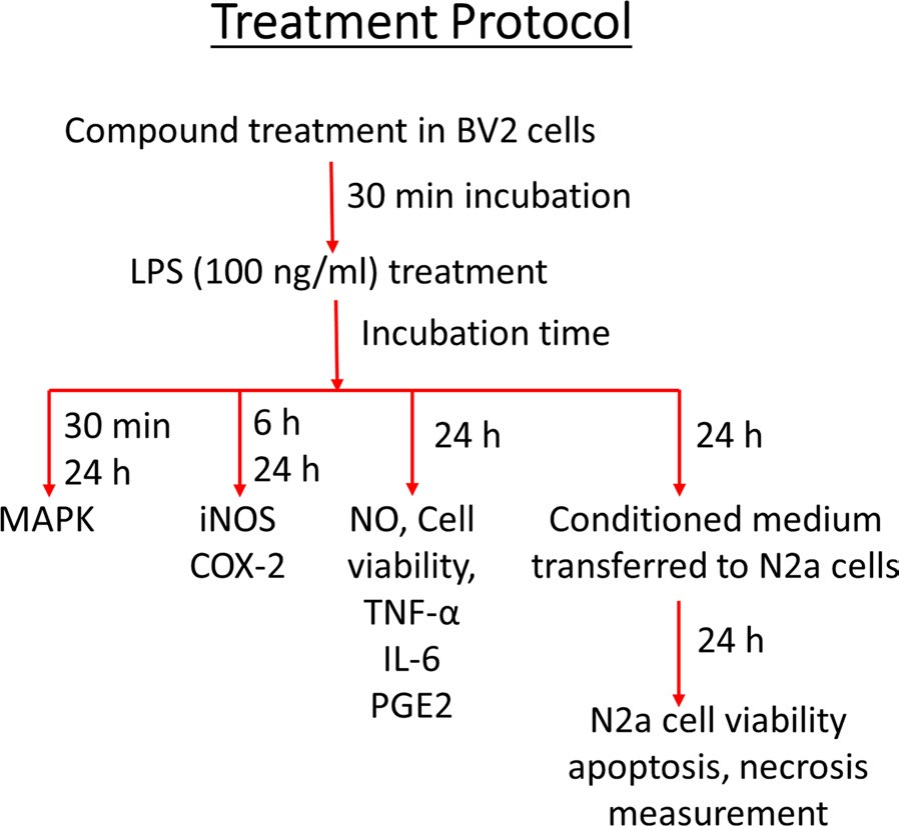
Treatment strategy for various parts of this experiment

The BV-2 microglial cell line was obtained as a gift from by Dr. E. Choi at Korea University (Seoul, Korea) and the murine neuroblastoma cell line (N2a) was originally obtained from American Type Culture Collection (Manassas, VA, USA). BV-2 and N2a cells were grown in uncoated tissue culture plates and incubated at 37 °C in a humidified atmosphere of 5% CO_2_ and 95% air. The cells were maintained in Dulbecco’s modified Eagle’s medium (DMEM) supplemented with 10% fetal bovine serum, 100 U/mL penicillin, and 100 U/mL streptomycin. The culture medium was changed every 24 h [26]. The overall treatment strategy for this experiment is shown in Scheme 2.

### Nitrite oxide and MTT assay

Nitrite oxide or MTT assay was performed as described by our previous publication with slight modification [27]. In order to measure MTT and NO production, BV-2 cells were plated into a 96-well plate at a density of 4 × 10^4^ cells/well. Seeded cells were pretreated with 5, 10, and 20 µM **compound 3** and incubated for 30 min. After the incubation, 100 ng/mL LPS was applied to all the wells with **compound 3**, except the untreated control group, and incubated for a further 24 h. Then, nitrite (a soluble oxidation product of NO) was detected in the culture media by using the Griess reaction. The supernatant (50 µL) was collected and mixed with an equal volume of Griess reagent (1% sulfanilamide in phosphoric acid and 0.1% *N*-1-napthylethylenediamine dihydrochloride). The absorbance was measured at 570 nm by using a microplate reader. Sodium nitrite was used to create a standard curve from which the NO_2_ concentration was calculated. The cell viability was evaluated by spectrophotometrically measuring the reduction of yellow 3-[4,5-dimethylthiazol-2-yl]-2,5-diphenyltetrazolium bromide (MTT) to purple formazan crystals. MTT was dissolved in DMEM, added to the culture plate containing cells at a final concentration of 0.5 mg/mL, and incubated for 1 h. The medium containing the MTT solution was carefully removed, and 200 µL DMSO was added to each well. After solubilizing the stained cells to produce a formazan-colored solution, the optical density (OD) was measured on a microplate reader at 570 nm, and the results were expressed as a percentage of the LPS-treated group. The same plate was used for the NO and MTT assays. The cell supernatant was used for the NO assay, and the plated cells were used for the MTT assay.

### ELISA

To measure TNF-α, IL-6, and PGE_2_, BV-2 cells were seeded in a 24-well plate at a density of 3 × 10^5^ cells/well. The cells were treated with LPS (100 ng/mL) in the presence or absence of **compound 3** for 24 h, and the media was collected and centrifuged [26]. IL-6, TNF-α, and PGE2 produced in microglial culture supernatants (inter-cellular) were measured by competitive enzyme immunoassay kits (R&D systems, Minneapolis, MN, USA) in accordance with the manufacturer’s protocol.

### Western blot analysis

BV-2 cells were seeded in a 6-well plate at a density of 6 × 10^5^ cells/well and exposed to LPS (100 ng/mL) in the presence or absence of **compound 3** for required time [26]. After treatment, cells were collected and lysed in cell lysis buffer (50 mM Tris–HCl, pH 8.0, 0.1% SDS, 150 mM NaCl, 1% NP-40, 0.02% sodium azide, 0.5% sodium deoxycholate, 100 µg/mL PMSF, and 1 g/mL aprotinin) for 24 h at 4 °C. The protein content was measured using a Bradford assay. Equal amounts of protein (30 µg) were separated using SDS-PAGE and transferred to a nitrocellulose membrane. The membrane was blocked for 1 h with 5% skim milk in tris-buffered saline with Tween-20 (TBST) and then incubated for 12 h with primary antibodies against α-tubulin (Sigma-Aldrich Cat. no: T5168), iNOS (Bioscience Cat. no: 610333, COX-2 (Santa Cruz Cat. no: sc-1745), ERK (Cell Signaling Cat. no: 9107s), pERK (Cell Signaling Cat. no: 5013s), JNK (Cell Signaling Cat. no: 9252s), pJNK (Cell Signaling Cat. no: 4671s), p38 (Cell Signaling Cat. no: 8690s), and pP38 (Cell Signaling Cat. no: 9211s), followed by incubation for 1 h with horseradish peroxidase-conjugated secondary antibodies (Cell Signaling) at 15 °C. The blots were developed using ECL Western Blotting Detection Reagents (Amersham Pharmacia Biotech, Buckinghamshire, UK). Densitometric analysis of the bands was performed using ImageMaster™ 2D Elite software (version 3.1, Amersham Pharmacia Biotech) [26].

### Annexin V/PI staining

An annexin V/PI apoptosis kit was used in accordance with the manufacturer’s instructions to quantify the percentage of cells undergoing apoptosis. First, BV-2 cells were incubated for 24 h with 5, 10, and 20 µM of **compound 3** or LPS (100 ng/mL). Then, N2a cells were treated with the supernatant of the treated BV-2 cells and incubated. After 24 h, the cells were collected, washed twice with cold PBS, and re-suspended in binding buffer at a concentration of 1 × 10^6^ cells/µL. Annexin V-FITC (5 µL) and PI (10 µL) were added, and the cells were incubated for 15 min at room temperature in the dark. Following incubation, 200 µL of binding buffer was added, and the cells were immediately analyzed by flow cytometry (FACSAriaIII; BD Biosciences, Franklin Lakes, NJ, USA). The flow cytometric analysis was performed using the Cell Quest software (BD Biosciences). The annexin V+/PI− cells and annexin V−/PI+ cells were used to identify apoptotic cells and necrotic cells, respectively. The procedure was repeated three times for each sample.

### Neuronal cell viability

N2a cells were used as a representative neuronal cell line for the examination of apoptotic neuron viability using direct treatment of compound 3 with TNF-α activation as well as conditioned media (CM) from LPS-stimulated BV-2 cells treated with **compound 3** [28]. This assay was performed following the methods described in our previous study, with slight modifications [26]. To measure neuron viability, BV-2 cells were first seeded and treated with 100 ng/mL LPS in the presence or absence of **compound 3**. After 24 h, the CM was collected and transferred to N2a cells for a further 24 h. The cell viability of N2a cells was measured using the MTT assay. Well differentiated neuronal cells were used for the experimental purpose as shown in Additional file 1: Fig. S1.

### Statistical analysis

The data were analyzed using Statistical Analysis System (SAS) software (PRISM). All data were expressed as the mean ± standard error of the mean. Statistical comparisons between different treatments were performed using one-way ANOVA followed by the Newman-Keuls post hoc test, using GraphPad Prism 5 (GraphPad Software Inc., La Jolla, CA, USA). Values of P < 0.05 were considered statistically significant. To confirm the reproducibility of the results, each experiment was performed in triplicate.

## Results

### Screening of effects of azine derivatives on LPS-induced NO production in BV-2 cells

We investigated whether azine derivatives had an inhibitory effect on the LPS-induced inflammatory response in BV-2 cells. The NO production and cell viability of LPS-activated BV-2 cells were measured after treatment with azine derivatives using Griess reagent and MTT assay, respectively [29]. Azine derivatives exhibited inhibitory effects on LPS-induced NO production and cell viability in BV-2 cells. As shown in Table 1, the azine derivatives suppressed LPS-induced NO production in BV-2 cells, except for **compound 4**. Among these derivatives, **compounds 6**, **7**, **8**, and **9** were cytotoxic to BV-2 cells at 20 µM. Since **compound 3** displayed an inhibitory effect on NO production in BV-2 cells without cytotoxicity or cell death, it was selected for the in vitro anti-neuroinflammatory study.

**Table 1:** Effects of azine derivatives on NO production in LPS-activated BV-2 cells

Compound	IC_50_^a^(µM)	Cell viability^b^(%)
Compound 1	58.52	90.33 ± 9.37
Compound 2	50.12	96.58 ± 1.75
*Compound 3*	*12.47*	*100.73* *±* *2.53*
Compound 4	> 500	91.03 ± 3.39
Compound 5	59.91	97.55 ± 10.59
Compound 6	47.36	87.90 ± 19.53
Compound 7	39.94	77.24 ± 5.67
Compound 8	47.89	69.45 ± 3.25
Compound 9	26.27	83.54 ± 7.78
L-NMMA^c^	18.32	100.40 ± 3.41

^a^The IC50 value of each compound was defined as the concentration (in µM) that caused 50% inhibition of NO production in LPS-activated BV-2 cells

^b^The cell viability was measured at 20 µM of compound treatment and it was expressed as a percentage (%) of the LPS-only treatment group. The results are the average of three independent experiments and the data are expressed as mean ± standard deviation

^c^L-NMMA was used as a positive control

### Effect of compound 3 on the LPS-induced neuroinflammatory response in BV-2 cells

**Fig. 2:**
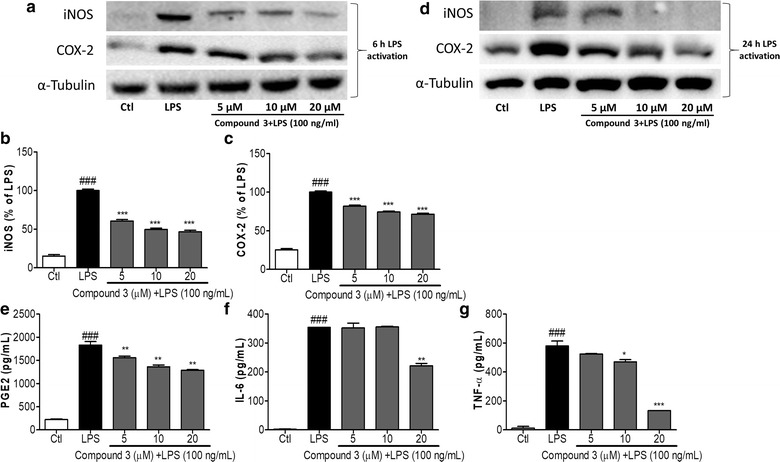
Effect of compound 3 on LPS-induced iNOS and COX2 expression in BV-2 cells. BV-2 cells were pretreated with various concentrations (5, 10, and 20 µM) of compound 3 for 30 min and activated with 100 ng/mL LPS. After 6 h, the cells were collected for analysis of iNOS and COX-2 expression using western blot analysis (**a**–**c**). Similarly, LPS activation was made for 24 h as chronic BV2 activation and the iNOS and COX-2 expression was measured (**d**). α-Tubulin was used as the loading control. The densitometric analysis of bands was performed as described in the methods section. After 24 h, the culture medium was subsequently collected to measure the quantity of PGE2 (**e**), IL-6 (**f**), and TNF-α (**g**) using ELISA analysis. All data are presented as the mean ± standard error of the mean of three independent experiments. *P < 0.05, **P < 0.01, ***P < 0.001 indicates significant differences compared with treatment with LPS alone while ^###^P < 0.001 indicates the significant differences compared with untreated control group. Here Ctl indicates untreated control and LPS indicates lipopolysaccharide

We investigated the inhibitory effect of **compound 3** on LPS-induced iNOS and COX-2 expression in BV-2 cells. As shown in Fig. 2a, pretreatment of BV2 cells with **compound 3** significantly reduced the expression of iNOS and COX-2 at all tested concentrations (5, 10, and 20 µM) (Fig. 2b, c). This effect was more potential even at the 24 h of chronic LPS activation. Compound 3 dramatically rescued BV2 cells against LPS induced iNOS and COX-2 activation as shown in Fig. 2d. Protein expression was normalized to the expression of α-tubulin, and the quantitative band intensity was calculated.

To investigate whether **compound 3** influenced the secretion of pro-inflammatory factors such as TNF-α, IL-6, and PGE2 in activated microglia cells, BV-2 cells were treated with **compound 3** for 30 min before LPS was added for 24 h. LPS-activated PGE2 production was reduced by **compound 3** at concentrations of 5, 10, and 20 µM in BV-2 cells (Fig. 2e). Similar results were obtained from the measurement of IL-6 and TNF-α secretion (Fig. 2f, g, respectively). Although 5 and 10 µM of **compound 3** did not significantly inhibit IL-6, there was noticeable activity occurred at 20 µM. Similarly, significant reduction of TNF-α secretion was observed at 10 and 20 µM in LPS-activated BV-2 cells. The Inter assay CV for all the elisa was confirmed less than 10% while the intra assay CV was less than 15% as shown in Additional file 2: Fig. S2.

### Effect of compound 3 on the MAPK pathway in LPS-induced BV-2 cells

**Fig. 3:**
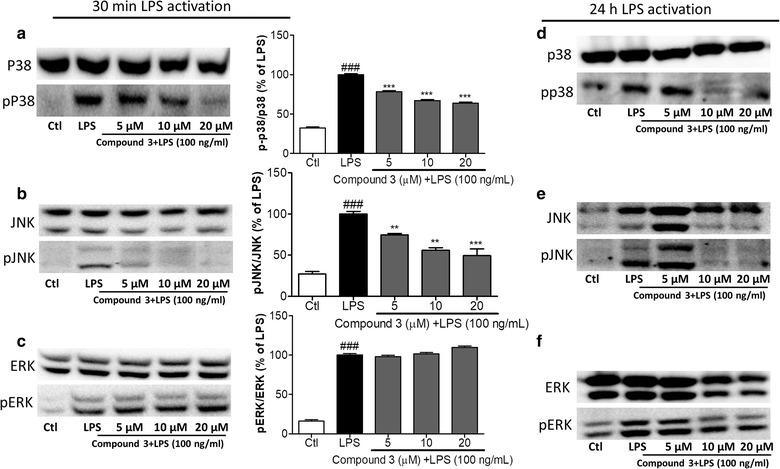
Effect of compound 3 on MAPK pathway in LPS-induced BV-2 cells. BV-2 cells were treated with LPS and with or without 5–20 µM compound 3 for 30 min. Cell lysates were prepared to evaluate the protein levels of MAPKs. The phosphorylation of p38, JNK, and ERK was measured by western blotting (**a**–**c**). Similarly, the cells were activated with LPS for 24 h as chronic activation and the expression pf MAPK was observed (**d**–**f**). The densitometric analysis of bands was performed as described in the methods section. **P < 0.01, ***P < 0.001 indicates significant differences compared with treatment with LPS alone; and ^###^P < 0.001 indicates significant differences compared with untreated control group. Here Ctl indicates untreated control and LPS indicates lipopolysaccharide

The mitogen-activated protein kinase (MAPK) signaling pathway plays an important role in controlling the synthesis and release of pro-inflammatory factors in LPS-stimulated BV-2 cells [30, 31]. Therefore, we investigated the effects of **compound 3** on MAPK activation in BV-2 cells. **Compound 3** significantly inhibited the LPS-induced phosphorylation of p38 as well as c-Jun *N*-terminal kinase (JNK) however, pretreatments of **compound 3** did not alter the phosphorylation of extracellular signal-regulated kinase (ERK) (Fig. 3). The pattern of activity is similar at short term LPS activation i.e. 30 min and chronic activation i.e. 24 h suggesting that, compound 3-mediated anti-inflammatory effect is through the inhibition of p38 and JNK phosphorylation.

### Effect of conditioned media (CM) treated with compound 3 on N2a cells

**Fig. 4:**
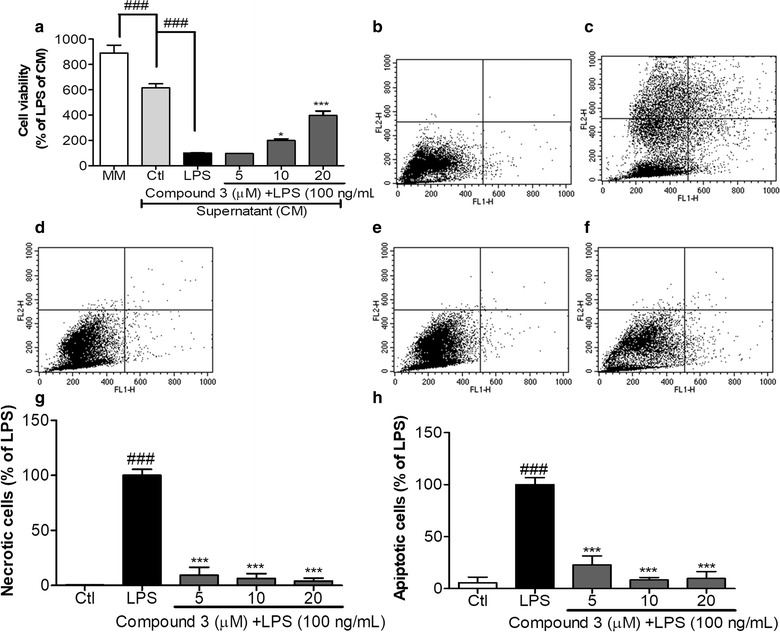
Neuroprotection of compound 3 in LPS-activated microglia in N2a cells. BV-2 microglial cells were pretreated with 5, 10, and 20 µM compound 3 for 30 min and stimulated with 100 ng/mL of LPS for 24 h. After 24 h of LPS treatment, the conditioned media (CM) were collected and transferred to N2a cells. After 24 h, MTT assay was done in the same CM treated N2a cells (**a**) and FACS analysis (**b**–**h**). An annexin V/PI apoptosis kit was used to quantify the percentage of cells undergoing apoptosis and necrosis (x-axis, annexin V; y-axis, PI) by using FACS analysis. CM-LPS treatment alone induced apoptosis and necrosis in N2a cells (**g**, **h**). All data are presented as the mean ± standard error of the mean of three independent experiments. *P < 0.05, ***P < 0.001; and ^###^P < 0.001 indicates significant differences compared with the CM-LPS group. Here Ctl indicates untreated control and LPS indicates lipopolysaccharide

Here, we investigated whether the CM of **compound 3** had any protective effects on neurons against activated microglia-induced MAPK signaling, NF-κB-mediated transcription, pro-inflammatory cytokines, and inflammatory mediators. N2a cell viability decreased when treated with CM containing only LPS (Fig. 4a). The CM of LPS induced significant cytotoxicity via the induction of apoptosis (Fig. 4b–h). The MTT assay showed that the CM of **compound 3** increased cell viability to 199.1 and 397.7% of the only LPS treated control value at concentrations of 10 µM and 20 µM, respectively. The CM of LPS induced apoptosis and necrosis in N2a cells, as determined by FACS analysis. As shown in Fig. 4g–h, treatment with LPS significantly increased the concentration of apoptotic and necrotic cells compared with BV-2 cells in normal conditions. However, the decrease in concentration of apoptotic and necrotic cells was mediated by the CM of **compound 3** at each concentration tested in N2a cells.

### Effect of compound 3 on cell viability of BV2 and N2a cells against LPS and TNF-α induced direct toxicity

**Fig. 5:**
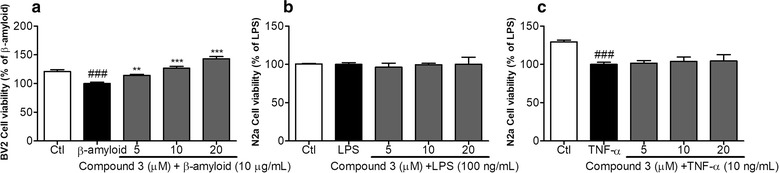
Cytoprotection of compound 3 in β-amyloid induced BV2 cell and LPS and TNF-α induced N2a cell toxicity

Here, we have investigated whether the **compound 3** has any direct protective effects on microglia and neurons against LPS and TNF-α induced direct toxicity. As the aim of this study is to check the effectiveness of compound 3 against neurodegenerative conditions, here we checked the effect of this compound to protect microglial cells against β-amyloid induced cytotoxicity as shown in Fig. 5a. Compound-3 significantly prevented microglial cell death against β-amyloid providing its cytoprotective effects. This result was further supported by the prevention of N2a cell survival against TNF-α induced N2a cytotoxicity (Fig. 5c). LPS treatment was unable to induce cellular toxicity to N2a cells (Fig. 5b). This results suggests that N2a cell death is not because of the LPS but because of the pro-inflammatory cytokines produced by the activated microglia. Compound 3 mediated neuroprotection in N2a cells is due to the inhibition of proinflammatory cytokines produced by LPS activated microglia.

## Discussion

The chronic activation of microglia is usually closely associated with neurodegenerative diseases. The neuroinflammatory response caused by the activation of microglia may induce neuronal cell death through its harmful pro-inflammatory cytokines. Activated microglia can induce production and secretion of many harmful pro-inflammatory mediators such as nitric oxide (NO), PGE2, TNF-α, and IL-6 [32]. Therefore, finding a potential candidate that can protect neurons against chronic microglia-mediated neurotoxicity is crucial for designing new therapeutic strategies for neurodegenerative disorders. According to previous reports, compounds that can activate autophagy could attenuate the level of iNOS, IL-6 and cell death in LPS activated microglial cells [33]. Azine compounds are known to regulate autophagy [34]. Lack of strong report on the neuroprotective effects of azine derivatives in microglia-mediated neurotoxicity inspired us to perform this experiment. We were to identify the most active azine derivative with anti-inflammatory activity, and to explore its acting mechanisms.

NO is an important inflammatory mediator that is affected by the activation of microglia [11]. Activated microglia can produce and secrete cytotoxic factors such as NO. NO is an important biomarker of neuroinflammation. Because of this reason, we performed nitrite assay for their anti-neuroinflammatory activity of azine and its derivatives. Most azine derivatives (except **compound 4**) inhibited the production of LPS-induced pro-inflammatory mediators such as NO, and demonstrated no cytotoxicity as shown in Table 1. However, **compounds 7** and **8** exhibited high toxicity in BV-2 cells. Among the derivatives tested, the **compound 3**, 4,4′-(1*E*,1′*E*,3*E*,3′*E*)-3,3′-(hydrazine-1,2-diylidene) bis(prop-1-ene-1-yl-3-ylidene) bis(2-methoxyphenol), strongly inhibited NO production without cytotoxicity. The inhibition was even greater than that of commercially available well-known *i*NOS inhibitor L-NG-monomethylarginine (L-NMMA) [35]. From this data we hypothesized that, the methoxyphenol substituent present in compound 3 made it the most potential candidate then that of other azine derivatives. It is believed that, a phenolic hydroxyl group has an important pharmachore potency. Since the azine derivative **3** substituted with a phenolic hydroxyl group showed the best activity, it is thought that the phenolic hydroxyl group is an important pharmacologically active group, which is consistent with the result that it exhibits various physiological activities. Therefore, our data may suggest that the presence of methoxyphenol and the substitution pattern within hydrazine derivatives influences the anti-neuroinflammatory activity. These results indicate that azine and its derivatives have the potential to alter the neuroinflammatory conditions induced by LPS activated BV2 microglia.

NO and PGE2 production are catalyzed by iNOS and COX-2, respectively, which are the key proteins in the regulation of inflammatory responses in the brain [15, 36]. As shown in Fig. 2, LPS-induced iNOS and COX-2 protein expressions were significantly inhibited by **compound 3**. The reduced expression of iNOS by **compound 3** might be caused by NO decrease in the same treatment group. Additionally, LPS-activated COX-2 expression was also lowered by **compound 3,** followed by the reduced production of PGE2 against LPS activated BV-2 cells.

The overproduction of pro-inflammatory cytokines has a harmful effect on microglia and neurons [37, 38]. They are believed to be a key player for the neurodegeneration as well as neuronal cell death. We tested **compound 3** for inhibition of TNF-α and IL-6 secretion in LPS-activated BV-2 cells. As shown in Fig. 2, treatment with **compound 3** suppressed the inflammatory response by decreasing the secretion of TNF-α and IL-6 in LPS-induced BV-2 cells. The inactivation of microglial cells by **compound 3** may be regulated by inflammatory cytokines such as IL-6 and TNF-α.

As LPS is a TLR4 agonist, once it binds to its receptor. And, it triggers the effector signaling pathways through the enhanced phosphorylation of MAPK proteins and followed by NF-κB mediated transcription [29]. With this notion, we hypothesized that the observed decrease in pro-inflammatory mediators were regulated by the MAPK pathway. To confirm this, we measured the expression of MAPKs by western blotting. **compound 3** downregulated the LPS-induced phosphorylation of p38 as well as JNK in a dose-dependent manner, but did not alter the expression of pERK. This mechanism of action suggests that **compound 3** non-specifically downregulated p-p38 and pJNK in LPS activated microglia. Studies suggested that the stress or endotoxin induced phosphorylation of p38 and JNK are major cause for the induction of apoptosis followed by the increased TNF-α secretion through NF-κB mediated transcription [27, 39]. And here we find that compound 3 inhibited the phosphorylation of p38 as well as JNK together with significant reduction of TNF-α. This result suggests that compound 3 might give protection against neuronal cell death induced by activated microglia. Furthermore, inhibitory effect of compound 3 on the p-p38 and pJNK was further confirmed in 24 h chronic LPS activation. In conclusion, the results of this study clearly provides evidences that **compound 3** has a potent anti-neuroinflammatory effect in LPS-activated microglia. This effect was achieved through the decreased expression of pro-inflammatory mediators via the suppression of the MAPK signaling pathways.

Chronic activation of microglia can produce several harmful pro-inflammatory factors through MAPK signaling and NF-κB mediated transcription. As a result, it may induce neuronal cell death through apoptosis [40]. As compound 3 inhibited MAPK signaling as well as proinflammatory cytokines, we hypothesized that it might prevent the neuronal apoptosis induced by LPS-activated microglia. Therefore, to prove this hypothesis, we investigated whether **compound 3** regulated the microglia-mediated neuronal cell toxicity. The conditioned medium (CM) of **compound 3** restored microglia-mediated neuronal cell toxicity to normal levels in N2a cells (Fig. 4), and no adverse effects were observed in either N2a or BV-2 cells. In addition, after exposure to **compound 3**, levels of apoptotic and necrotic cells were returned to normal, which was attributed to the downregulation of pro-inflammatory factors by the CM of **compound 3** in N2a cells. As is confirmed in our experiment, *B*-amyloid can induce microglia activation as well as microglial death. Compound 3 protected against *B*-amyloid-induced BV2 cells toxicity (Fig. 5). For further clarification of mode of action, TLR4 expression [27] was measured to answer whether the toxicity was due to LPS itself or pro-inflammatory cytokines. To answer this question, we treated N2a cells with LPS and TNF-α directly. We found that direct LPS treatment does not induced N2a cell death where it was significantly induced with TNF-α treatment (Fig. 5b, c). In support of earlier results, **compound 3** increased cell viability of N2a cells against TNF-α mediated toxicity. As a result, we can conclude that TNF-α produced by LPS activated microglia is responsible for N2a cell death. Inhibition of TNF-α production by compound 3 is one of the major action mechanisms giving protection of neurons. Namely, compound 3 protected against activated microglia induced N2a cell toxicity. This result suggests that **compound 3** may protect neuronal cell damage via inhibition of the pro-inflammatory cytokine released from activated microglia through inhibition of p38 and JNK phosphorylation. Further experiments are required to study the potential effect of **compound 3** on toxin-mediated neuronal cell damage specially in vivo experiments.

## Conclusions

**Fig. 6:**
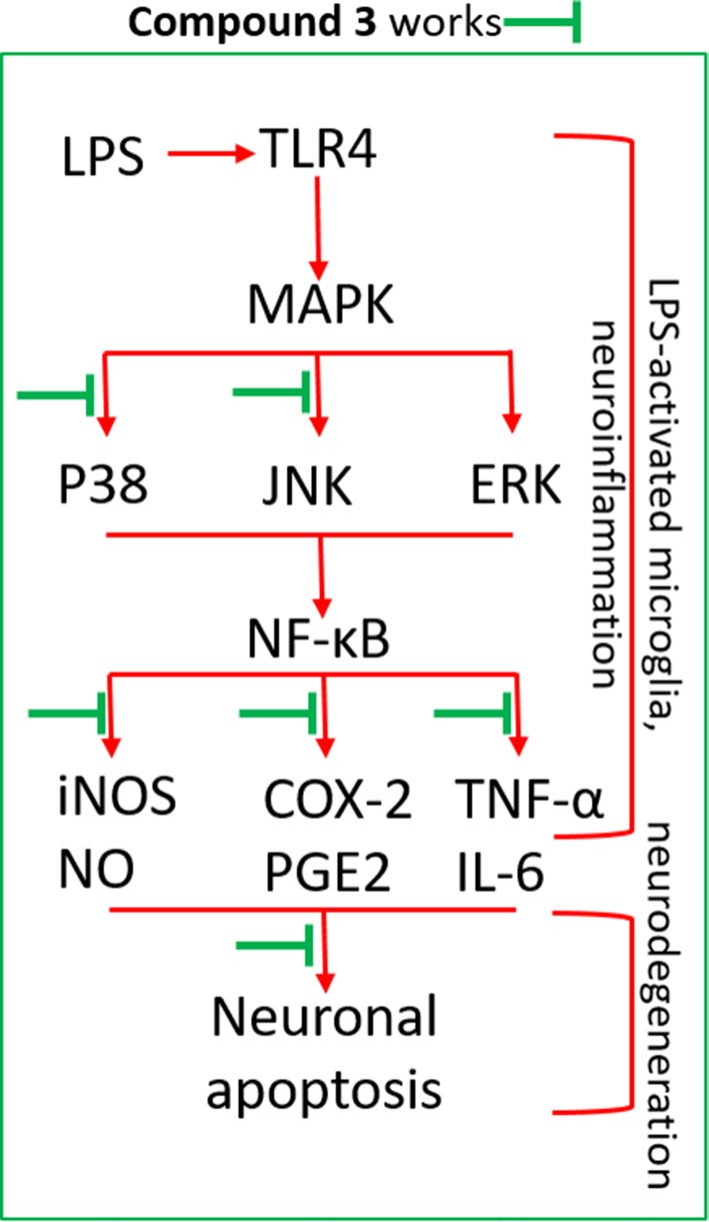
Summary of the effect of compound 3 on neuroinflammation and neurodegeneration inhibition

As shown in the summary Fig. 6, we have demonstrated for the first time, the key role of the highly conjugated azine derivative **compound 3**, in the regulation of activated microglia for the protection of neuronal cells. **Compound 3** modulated over activated microglia by downregulating the MAPK pathway (specially p-p38 and pJNK) and pro-inflammatory factors (such as TNF-α, IL-6, NO, and PGE2) by the regulation of iNOS and COX-2 expression in acute as well as chronic LPS-stimulated BV-2 cells. In addition, **compound 3** protected N2a cells against microglia-mediated neurotoxicity. Our findings validated the effectiveness and safety (low toxicity) of **compound 3** as a therapeutic neuroprotective agent which can attenuate chronically activated microglial inflammation. Further in vivo studies are needed to confirm its efficacy and safety.

## Additional files

**Additional file 1: Fig. S1.** Differentiation of N2a cells after serum starvation. N2a cells were treated in serum free condition, that let cells for proper differentiation and with the treatment of LPS, degeneration of neurite outgrowth can be seen.

**Additional file 2: Fig. S2.** Inter and intra assay CV for all the Elisa performed in this experiment.

## Abbreviations

CNS: central nervous system
AD: Alzheimer’s disease
PD: Parkinson’s disease
LPS: lipopolysaccharide
IFN-γ: interferon-γ
NO: nitric oxide
PGE2: prostaglandin E2
IL-6: interleukin-6
IL-1β: interleukin-1β
TNF-α: tumor necrosis factor-α
iNOS: inducible nitric oxide synthase
COX-2: cyclooxygenase 2
ROS: reactive oxygen species
MTT: 3-[4,5-dimethylthiazol-2-yl]-2,5-diphenyltetrazolium bromide
OD: optical density
NMMA: N-monomethyl-l-arginine
