# Supplementary figures and images for: N,N-disubstituted azines attenuate LPS-mediated neuroinflammation in microglia and neuronal apoptosis via inhibiting MAPK signaling pathways
Source: BMC Neurosci. 2017 Dec 28;18:82. doi: 10.1186/s12868-017-0399-3 (PMC5745756; doi:10.1186/s12868-017-0399-3)

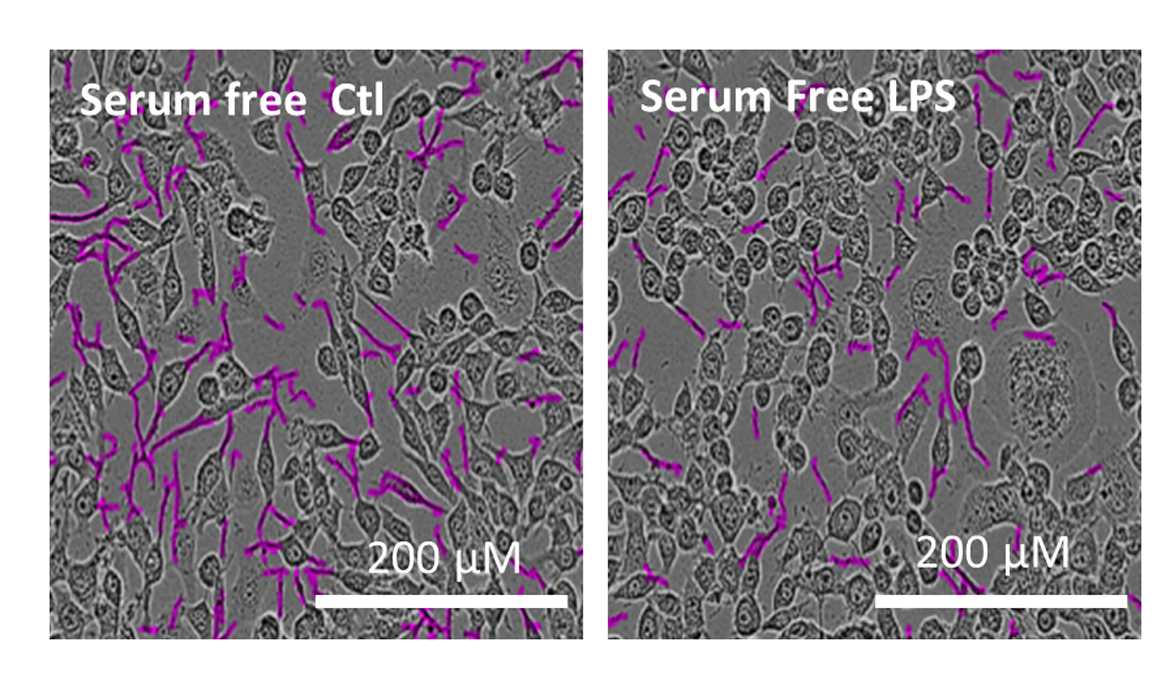

Supplement: Supplementary file 1 — Additional file 1: Fig. S1. Differentiation of N2a cells after serum starvation. N2a cells were treated in serum free condition, that let cells for proper differentiation and with the treatment of LPS, degeneration of neurite outgrowth can be seen. [file 12868_2017_399_MOESM1_ESM.tif]

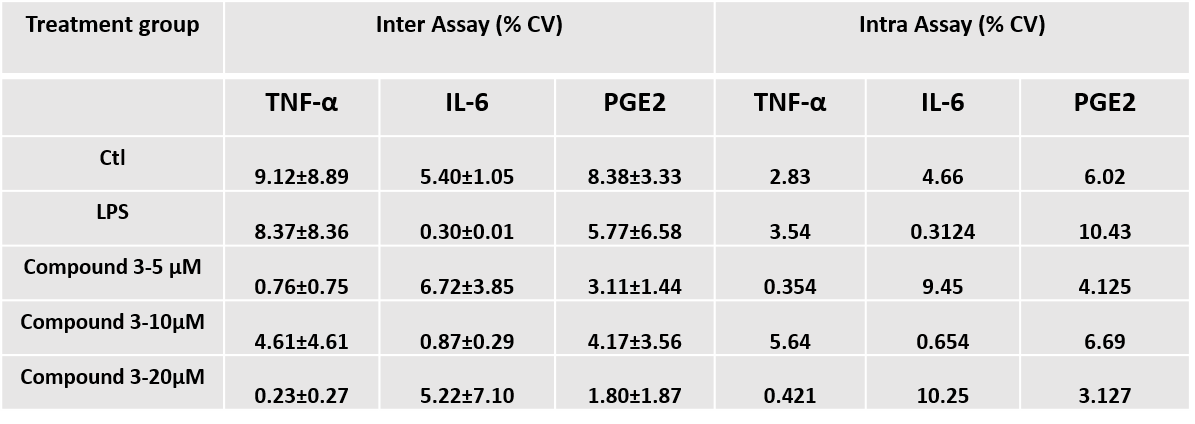

Supplement: Supplementary file 2 — Additional file 2: Fig. S2. Inter and intra assay CV for all the Elisa performed in this experiment. [file 12868_2017_399_MOESM2_ESM.tif]
